# Diagnostics of *IDH1/2* Mutations in Intracranial Chondroid Tumors: Comparison of Molecular Genetic Methods and Immunohistochemistry

**DOI:** 10.3390/diagnostics14020200

**Published:** 2024-01-16

**Authors:** Vyacheslav Varachev, Anastasia Shekhtman, Dmitrii Guskov, Dmitrii Rogozhin, Alexander Zasedatelev, Tatiana Nasedkina

**Affiliations:** 1 Engelhardt Institute of Molecular Biology, Russian Academy of Sciences, 119991 Moscow, Russia; varachevviacheslav95@mail.ru (V.V.); dimguskov@mail.ru (D.G.); zas@biochip.ru (A.Z.); 2 N.N. Burdenko National Medical Research Center of Neurosurgery, Ministry of Health of the Russian Federation, 125047 Moscow, Russia; ektozz@gmail.com; 3 Russian Children’s Clinical Hospital, N.I. Pirogov Russian National Research Medical University, Ministry of Health of the Russian Federation, 119571 Moscow, Russia; pathol.777@mail.ru; 4 N.N. Blokhin National Medical Research Center of Oncology, Ministry of Health of the Russian Federation, 115522 Moscow, Russia

**Keywords:** intracranial chondroid tumors, *IDH1/2* mutations, diagnostics, immunohistochemistry, biochip assay, DNA melting analysis, Sanger sequencing

## Abstract

Intracranial chondroid tumors are a heterogeneous group of neoplasms characterized by the presence of a cartilage matrix. These tumors exhibit overlapping clinical and histological features. Mutations in *IDH1/2* genes serve as important diagnostic markers of tumor type, particularly chondrosarcoma. To improve the accuracy of *IDH1/2* diagnostics, we compared three methods: biochip assay, real-time PCR with DNA melting analysis using TaqMan probes and sequencing (qPCR-DMA-Sanger), and immunohistochemistry (IHC). Tumor samples from 96 patients were investigated. The *IDH1* mutations were detected in 34/64 (53%) chondrosarcomas; IHC detected 27/56 (48.2%) mutations, the qPCR-DMA-Sanger method 27/59 (46%) mutations, and the biochip assay revealed 29/60 (48.3%) mutations. The detection of *IDH1* mutations in chordoma (2/15) and osteosarcoma (2/7) suggested the need for a revised diagnosis. In benign tumors, *IDH1* mutations were present in chondroma (4/6), but absent in chondromyxoid fibroma (0/4). The most frequent *IDH1* mutations were R132C (60%), R132L, and R132G (13.5% each), R132H (8%), and R132S (5%). The concordance between the biochip assay and IHC was 90%, between IHC and PCR-DMA-Sanger 83%, and between biochip assay and qPCR-DMA-Sanger was 98%, respectively. No *IDH2* mutations were found. The use of independent diagnostic methods may improve the detection of *IDH*-mutant specimens in chondroid tumors.

## 1. Introduction

Mutations in the genes of isocitrate dehydrogenases 1 and 2 (IDH1 and IDH2) play an essential role in the development of a number of tumors (gliomas, chondroid tumors, leukemia), and are important for the diagnosis and choice of therapy [1,2,3]. Isocitrate dehydrogenases are involved in the oxidative decarboxylation of isocitrate, converting it into α-ketoglutarate, an intermediate product of the Krebs cycle. Somatic mutations in the *IDH1* and *IDH2* genes are heterozygous nucleotide substitutions in the catalytic domain, leading to a loss of the normal functional activity of the enzyme, reduction of α-ketoglutarate levels, and formation of 2-hydroxyglutarate, which has oncogenic activity [3,4,5]. Mutations in the *IDH1* gene occur in codon R132, with >90% of mutations in gliomas associated with arginine to histidine (p.R132H) substitution, while a wider range of amino acid changes have been observed in chondroid tumors [1,6,7,8]. In the *IDH2* gene, mutations affect the R172 codon, with arginine to lysine (p.R172K) being the most frequent substitution [9]. Mutations in the *IDH1* gene are much more common than mutations in the *IDH2* gene and account for >95% of all cases of the *IDH1/2* mutant genotype [3,6,8]. 

Intracranial chondroid tumors (ICHTs) are rare neoplasms, comprising, according to different data, from 0.15 to 2% of all head and neck tumors [10]. All ICHTs can be divided into two groups depending on the nature of the cartilage matrix. The first group is cartilaginous matrix tumors, which include chondromas and chondrosarcomas, as well as the chondroblastic variant of conventional osteosarcoma. The second group represents tumors containing mostly cartilage-like or myxoid matrix: chondroid chordoma, chondromesenchymal hamartoma, and chondromyxoid fibroma. Depending on the degree of malignancy, ICHTs can be divided into benign (chondroma, chondromesenchymal hamartoma, chondromyxoid fibroma), and malignant (chordoma, chondrosarcoma, chondroblastic osteosarcoma) tumors [11,12]. 

Intracranial chondrosarcomas represent a very heterogeneous group of ICHTs and are classified into several subtypes: conventional chondrosarcoma, clear cell chondrosarcoma, mesenchymal chondrosarcoma, and dedifferentiated chondrosarcoma. Conventional chondrosarcomas represent the most common pathological entity among all chondrosarcomas (80%) and, in turn, can be divided according to their bone location in central and peripheral chondrosarcomas [11,13]. In addition, the conventional chondrosarcomas are classified as primary or secondary if they arise de novo or from pre-existing lesions (osteochondroma, enchondroma) [11,12,14]. 

Regardless of the histological variant, ICHTs are most often localized in bones of the skull base, namely in the region of spheno-occipital synchondrosis [10]. The most common clinical manifestations of ICHTs are headache and oculomotor disorders associated with lesions of the third cranial nerves. The strategies of treatment and prognosis differ significantly between benign and different malignant types, while preoperative diagnostics is a difficult complex task [14]. The molecular analysis of clinically relevant diagnostic markers, exemplified by *IDH1/2* mutations, can significantly improve decision-making in patient management. The hot-spot *IDH1/2* mutations have been found to be hallmarks of several types of chondroid tumors and are mainly encountered in benign enchondromas (up to 87%) [7], central conventional chondrosarcomas (about 50%), and dedifferentiated chondrosarcomas [6,15].

The accuracy and reproducibility of the methods used to detect *IDH1/2* mutations are important for implementing molecular findings into medical practice guidelines. Direct sequencing seems to be the “gold” standard in assessing the mutational status of *IDH1/2* genes [16]. However, the sensitivity of this method depends on the quality of the sample; moreover, the threshold for the detection of mutant DNA against wild-type DNA is above 10% [16,17]. 

Another widely used method for detecting mutations in *IDH1/2* genes is immunohistochemistry (IHC) using mono- or polyclonal antibodies, which is considered a fast, reliable, and cost-effective laboratory method [16,17]. At the same time, IHC is sensitive to the quality of the sample and requires the development of specific antibodies for the detection of mutation type. Also, the heterogeneity in staining of tumor specimens and cross-reactivity of antibodies may be observed [17,18,19]. 

Such methods of detecting *IDH* mutations as allele-specific PCR [20] or pyrosequencing [21] are used in clinical practice, but they have no significant advantages over traditional sequencing. The use of asymmetric real-time PCR in combination with DNA melting analysis using a TaqMan probe (qPCR-DMA) was proposed to detect somatic mutations with a sensitivity of 5%, but Sanger sequencing is additionally required to identify nucleotide substitutions [22]. 

The use of next-generation sequencing (NGS) allows identification of variant alleles at frequencies of 0.1% and lower [23], which may substantially increase the sensitivity of *IDH1/2* genotyping. In addition to *IDH1/2* hot-spot mutations, a complex mutational landscape including deleterious variants in *TP53, EGFR, APC,* and *ATM* genes has been revealed in chondrosarcomas [24]. However, this approach requires rather expensive equipment, sophisticated data analysis, highly qualified personnel, and a sufficient amount of tumor material; all of these things together probably make it not so cost-effective when examining a very limited number of targets. 

Another diagnostic tool used in routine diagnostics is biological microarrays (biological microchips, biochips) [25]. It was shown previously that biochips can be used for the analysis of somatic mutations in different types of cancer (lung cancer, melanoma, colorectal cancer) [26]. The biochip-based approach was developed for the simultaneous analysis of mutations in the *IDH1* and *IDH2* genes and the ability to detect different *IDH1/2* mutations was demonstrated in control samples [27]. 

In the present study, we applied wild-type blocking PCR with the use of locked nucleic acid (LNA) oligonucleotides to increase the sensitivity of the biochip assay for *IDH1/2* mutations analysis. One aim of the study was to test this high-sensitive approach on a collection of chondroid tumor samples and to compare it with other methods: qPCR-DMA followed by Sanger sequencing and IHC. Another important aim was to determine the *IDH* mutational status in a series of 96 intracranial chondroid tumors, including chondrosarcomas, chordomas, osteosarcomas, chondromas, and chondromyxoid fibromas in order to evaluate the diagnostic utility of *IDH1/2* mutations for these types of neoplasms. 

## 2. Materials and Methods

### 2.1. Study Cohort

The retrospective study included ICHTs that were diagnosed between 2012 and 2019 in different Russian medical centers, mainly in the N.N. Burdenko National Medical Research Center of Neurosurgery (Moscow). The slides and paraffin blocks were centralized and reviewed at the Pathology Department of the Russian Children’s Clinical Hospital (N.I. Pirogov Russian National Research Medical University, Moscow). 

Finally, we collected samples from 96 patients with ICHTs (96 surgical resections) ranging in age from 2 to 74 years (mean age 39.6 years), 29 males and 67 females. The diagnostic criteria based on morphological, radiological, and histological data, were applied as described earlier [28]. The diagnoses between the 96 cases were categorized as follows: chondrosarcoma (*n* = 64), chordoma (*n* = 15), osteosarcoma (*n* = 7), chondroma (*n* = 6), and chondromyxoid fibroma (*n* = 4). 

All tissue samples were formalin-fixed and paraffin-embedded (FFPE). Before embedding, the decalcification stage was performed with the use of special decalcifying solutions (10% formic acid or 5% hydrochloric acid). After preparation, the FFPE blocks were used for histological analysis, immunohistochemical staining, and molecular genetic analysis.

### 2.2. DNA Isolation

DNA was extracted from FFPE tissue using QIAamp DNA FFPE (Qiagen GmbH, Hilden, Germany) according to the manufacturer’s protocol. Concentration and purity (260/280 nm ratio) of DNA were determined by using the NanoDrop spectrophotometer (Thermo Fisher Scientific, Waltham, USA). 

### 2.3. Real-Time PCR and DNA Melting Analysis Using TaqMan Probes and Sanger Sequencing (qPCR-DMA-Sanger)

Mutations were analyzed by asymmetric real-time PCR followed by melting analysis of the PCR product in the presence of TaqMan probes, as described previously [27]. Detection of mutations in the *IDH1* and *IDH2* genes was performed in different tubes. The primer and probe sequences are in Table S1.

The 25µL of PCR mixture contained 50 mM Tris-HCl, pH 8.8, 50 mM KCl, 0.01% (*v*/*v*) Tween 20, 3 mM MgCl2, 0.25 mM dNTPs, 1.25 U Taq polymerase, a primer pair (0.04 µM: 0.4 µM), 0.2 µM TaqMan probe (DNA Synthesis, Ltd., Moscow, Russia), and 5 µL of DNA template.

Briefly, the reaction was performed in a LightCycler 96 amplifier (Roche, Diagnostics, Rotkreuz, Switzerland) for both primer pairs: 95 °C for 5 min, then (95 °C for 13 s, 57 °C for 40 s, 72 °C for 20 s) × 53 cycles; melting of PCR products: 95 °C for 1 min, 55 °C for 4 min, then from 55 to 90 °C, increasing the temperature by 0.2 °C for each step with a step duration of 12 s. Further, samples positive for *IDH1/2* mutations were sequenced on an Applied Biosystems 3730 DNA Analyzer (Applied Biosystems, Waltham, USA) using the standard protocol.

### 2.4. Biochip Manufacturing

The biochip for the detection of *IDH1* and *IDH2* mutations and the sequences of immobilized oligonucleotides were described earlier [27]. Briefly, oligonucleotide probes 14–20 bp in length were synthesized on a 394 DNA/RNA synthesizer (Applied Biosystems, USA) using standard phosphoramidite chemistry. The oligonucleotides carry an amino group at the 3’-terminus for immobilization in the polyacrylamide gel drops on a biochip using a copolymerization method [25]. 

### 2.5. Wild-Type Blocking PCR and Hybridization with Biochip

To obtain a single-stranded and fluorescently labeled DNA fragment for hybridization on the biochip, a two-step nested PCR method was used as previously described [26,27]. In the first step, the wild-type blocking PCR with LNA probes was used. The sequences of primers and LNA probes are in Table S2. The concentration of LNA oligonucleotide was 0.02–0.2 µM for 5–10 ng of genomic DNA in the reaction. During the first step, a double-stranded product was generated, predominantly from mutant DNA. In the second step, the product of the first step was used as a matrix and asymmetric PCR was performed with simultaneous labeling with fluorescent Cy5-dUTP of the single-strand PCR product. Hybridization of the fluorescently labeled PCR product on a biochip was performed under the conditions described previously [26,27]. Fluorescent signals were registered using a biochip analyzer, and image analysis and genotype assignment were performed using the ImaGeWare ver.3.5 software (Biochip-IMB, Ltd., Moscow, Russia) [25,26]. 

### 2.6. Immunohistochemistry (IHC)

The 4 mm thick tissue sections were cut, heated at 58 °C for 2 h, deparaffinized, and immunostained on a fully automated Roche Ventana Bench-Mark Ultra system (Roche Diagnostics, Rotkreuz, Switzerland) with subsequent hematoxylin staining following the manufacturer’s guidelines. The tissue samples underwent immunostaining using specific antibodies, including a rabbit polyclonal anti-IDH1 R132 mutation antibody, a mouse IgG2b/K monoclonal antibody for brachyury, monoclonal antibodies for S100 protein, epithelial membrane antigen (EMA), and Ki-67 (Invitrogen, Thermo Ficher Scientific, Waltham, MA, USA).

## 3. Results

### 3.1. Clinical Characteristics

The basic criteria for patient inclusion in the study involved the presence of true cartilage or cartilage-like (chondroid) matrix in the tumor tissue and anatomical localization of the pathological process in the cranial bones. The histologic features and the clinical data available are in Table 1.

Malignant tumors were represented by chondrosarcoma (*n* = 64), chordoma (*n* = 15), and osteosarcoma (*n* = 7). Chondrosarcomas (*n* = 64) constituted the largest group. The S100 protein expression was detected in the vast majority of cases (96%). The following histologic subtypes were identified: primary central chondrosarcoma/atypical cartilaginous tumour (ACT), grade 1 (29.7%); primary central chondrosarcoma, grades 2 (65.6%) and 3 (1.6%); and dedifferentiated chondrosarcoma (3.1%). The most frequent tumor process involved the cavernous sinus (43%), sphenoid bone (40%), clivus (34%), temporal bone (34%), and ethmoid bone (25%). More rarely, the tumor process spread to the facial skeleton (15%), dura mater (9%), and falx cerebri (4%).

Among the chordoma specimens (*n* = 15), the majority belonged to the classical variant (*n* = 8, 53%), and one third (*n* = 5, 33%) was represented by the chondroid variant of chordoma with the presence of cartilaginous matrix. In one case, a chordoma combined with an aneurysmal bone cyst (ABC) was observed. Most chordomas involved the clivus (77%), sphenoid bone (54%), and cavernous sinus (38%). In 85% of cases, an expression of a specific brachyury protein was detected in the tumor cells. 

Osteosarcoma specimens (*n* = 7) were represented by osteosarcoma, not otherwise specified (NOS) (*n* = 3), chondroblastic variant osteosarcoma (*n* = 3), and undifferentiated pleomorphic osteosarcoma (*n* = 1). Osteosarcomas involved various bones of the skull: cavernous sinus, sphenoid bone, ethmoid bone, and also orbit, maxillary sinuses, and nasopharynx.

Benign intracranial tumors were represented by chondroma (*n* = 6) and chondromyxoid fibroma (*n*= 4). Most cases of chondroma affected the superior sagittal sinus (*n* = 4) and falx cerebri (*n* = 3).

### 3.2. Detection of IDH1/2 Mutations Using qPCR-DMA Followed by Sanger Sequencing 

**Figure 1:**
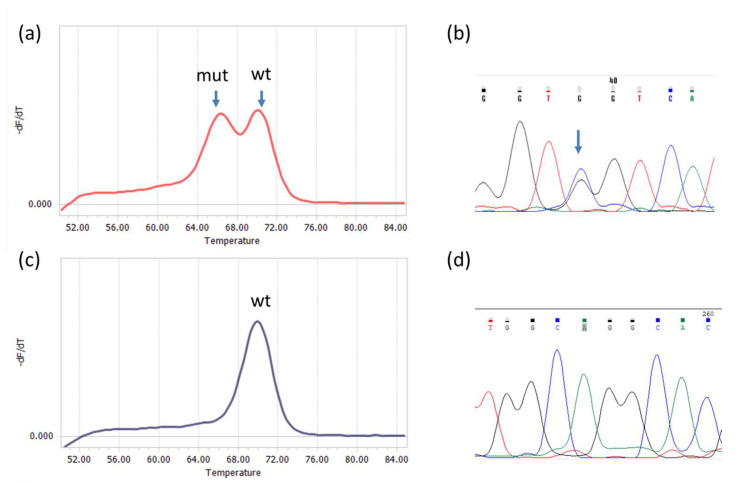
Detection of R132 *IDH1* and R172 *IDH2* mutations by qPCR−DMA−Sanger; (**a**) melting curve for the *IDH1* gene, one peak corresponds to a homoduplex of wild−type DNA with the TaqMan probe (wt), the other represents a heteroduplex of mutated DNA with the TaqMan probe; (**b**) sequencing of the PCR product shows the presence of CGT>GGT substitution in codon R132; (**c**) melting curve for the *IDH2* gene with only one peak corresponding to wild−type DNA (wt); (**d**) the result was confirmed by wild-type sequence AGG in codon R172.

Analysis was performed for *IDH1* and *IDH2* mutations independently. All 96 tumor samples were screened for *IDH1* mutations, but due to an insufficient amount of the material, only 79 were analyzed for *IDH2* mutations. The sizes of *IDH1* and *IDH2* PCR products were 143 bp and 148 bp, respectively. Examples of melting curves and sequences obtained for the PCR products are shown in Figure 1.

### 3.3. Detection of IDH1/2 Mutations Using a Biochip Assay

**Figure 2:**
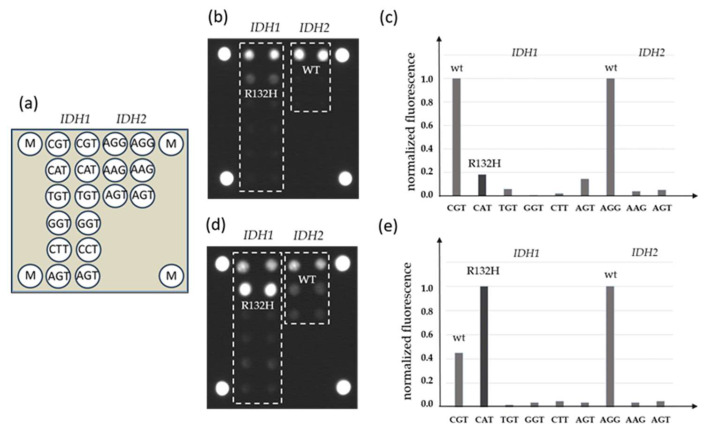
Detection of *IDH1* and *IDH2* mutations by a biochip assay; (**a**) scheme of the biochip with immobilized probes; (**b**) the hybridization pattern without inhibition by LNA oligonucleotides and (**c**) levels of normalized fluorescent signals from biochip cells; (**d**) hybridization pattern after inhibition of wild-type DNA amplification by LNA oligonucleotides and (**e**) levels of normalized fluorescent signals from biochip cells, the *IDH1* mutation R132H is revealed (M—fluorescent marker).

DNA samples from FFPE blocks were used in parallel for qPCR-DMA-Sanger and biochip assay. A combination of two-step nested PCR and hybridization analysis with immobilized oligonucleotide probes was used in the biochip assay (Figure 2).

To increase the sensitivity of the assay, LNA oligonucleotides complementary to the *IDH1* or *IDH2* wild-type sequence in the corresponding position were added to the PCR mixture during the first step of the reaction. 

It was shown previously that the wild-type DNA blocking during amplification could significantly increase the sensitivity of the assay up to 1% of mutated DNA against the 99% wild-type DNA background [26].

### 3.4. Detection of IDH1 mutations using IHC

**Figure 3:**
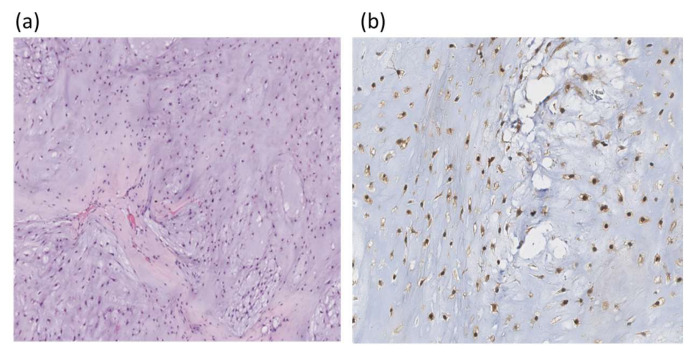
Histologic section of chondrosarcoma; (**a**) hematoxylin and eosin staining ×100; (**b**) IHC with polyclonal IDH1 antibodies, the nuclear reaction is observed (×200).

Using IHC, only *IDH1* mutations were tested. An example of IHC staining of chondrosarcoma histological section with an IDH1 polyclonal antibody is in Figure 3. 

### 3.5. IDH1/2 Mutational Status of Intracranial Chondroid Tumors

The 96 archived FFPE samples of ICHTs (chondrosarcoma, chordoma, osteosarcoma, chondroma, and chondromyxoid fibroma) were tested for the presence of *IDH1* mutations using three methods: biochip assay, qPCR-DMA with the TaqMan probe followed by sequencing of IDH1-positive samples, and IHC with IDH1-specific polyclonal antibodies. 

In chondrosarcomas, mutations in the *IDH1* gene were detected at least by one method in 34/64 (53%) samples. The biochip assay identified 29/60 (48.3%) mutations, the qPCR-DMA with sequencing detected 27/59 (46%), and the IHC method could reveal 27/56 (48.2%) mutations. The diagnostic yield of all three methods together was 21/47 (45%) mutations (Table 2).

The *IDH1* mutations were also found in 2/15 chordomas; both samples positive for the *IDH1* mutation histologically were diagnosed as chondroid chordoma. In one case (37 CRD), the *IDH1* mutation was detected by two methods: qPCR-DMA-Sanger and IHC; no brachyury expression was found in the sample. In the case of 38 CRD, the *IDH1* mutation was revealed by both molecular methods, while the IHC method was not applicable to this sample due to the small quantity of tumor material (Table 3).

In osteosarcoma patients (*n* = 7), two samples were found to be positive for the *IDH1* mutation; both cases were diagnosed as a chondroblastic variant of osteosarcoma. In one case, 35 CHOS, the *IDH1* mutation was detected only by qPCR-DMA-Sanger and in the other case, 36 CHOS, the mutation was revealed only by IHC.

The *IDH1* mutations were also detected in 4/6 (66%) chondroma samples (two mutations found by IHC and one of the molecular methods, while two mutations were revealed by one of the molecular methods). No *IDH1* mutations were found in chondromyxoid fibroma (*n* = 4).

The summary of *IDH1* mutation findings using different approaches is in Table 4.

Among *IDH1* mutations in the total sample, TGT was the most frequently detected codon at 59.5%, CTT and GGT at 13.5% each, CAT at 8%, and AGT at 5%. In chondrosarcomas, this proportion between different mutations was practically the same: TGT at 60%, CTT and GGT at 17% each, CAT at 3%, and AGT at 3% (Table 5). 

The concordance in mutation analysis between the different methods was calculated (Table 6). Between the biochip assay and IHC, the percentage of matched cases was 90%; between qPCR-DMA-Sanger and IHC, it was 83%; and between the biochip assay and qPCR-DMA-Sanger, it was 98%, respectively.

The mutations in the *IDH2* gene were analyzed only by two molecular methods: qPCR-DMA-Sanger and biochip assay. In summary, the testing for *IDH2* mutations was applicable to 79 samples of ICHTs and no *IDH2* mutations were found.

## 4. Discussion

Our study is primarily aimed at validation of a novel biochip-based approach to diagnose *IDH1* and *IDH2* mutations by comparing with known methods such as IHC and real-time PCR and DNA melting analysis using TaqMan probes combined with Sanger sequencing (Figure 1, Figure 2 and Figure 3). The methods were applied to study a sample of 96 ICHTs localized predominantly at the base of the skull (Table 1). There are few available studies on the frequency of *IDH1/2* mutations in cartilage tumors of this rare localization, and they mainly concern chondrosarcomas [8,29,30,31,32]. The *IDH1/2* mutation rate in different histological tumor types provides us with important insights into the clinical utility of *IDH1/2* diagnostic markers and pathways of malignant neoplasms.

The main problem in mutation analysis of cartilaginous tumors is the aggressive processing of the investigated material during the mandatory decalcification procedure; the pre-treatment prior to embedding into paraffin may be the most common cause of failed results [33]. In our study, the IHC method was the most sensitive to aggressive decalcification and was successful in only 84/96 (88%) samples, whereas the biochip assay was able to analyze 89/96 (93%) tumor samples, and qPCR-DMA-Sanger 88/96 (92%), respectively. Nevertheless, the detection rate of the *IDH1* mutation was almost similar for all three methods in the whole sample of 96 tumors (36%, 36%, and 37%, see Table 4), but in chondrosarcomas, qPCR-DMA-Sanger showed a slightly lower sensitivity (46%) compared to biochip (48.3%) or IHC (48.2%) methods.

The higher efficiency of IHC compared to conventional Sanger sequencing has been previously demonstrated [19]. Insufficient sensitivity of Sanger sequencing in detecting mutations present in less than <30% of the sequenced PCR product may lead to false negative results in samples with a low frequency of *IDH*-mutant alleles. Incorrect assignment of genotype, in turn, can be a source of error in evaluating the prognostic significance of *IDH* mutations [13]. 

High-resolution melting analysis can be used to detect *IDH1/2* mutations, which is less costly and time-consuming, yet more sensitive than Sanger sequencing [22,34]. In our study, we used a combination of asymmetric real-time PCR and TaqMan probe melting assay (qPCR-DMA) with Sanger sequencing of *IDH*-positive samples to increase the efficiency of analysis, validate the findings, and identify the nucleotide substitution. In one case, we discovered the *IDH1* mutation by qPCR-DMA, but direct sequencing revealed an *IDH* wild-type genotype. 

The concordance between biochip assay and IHC analysis was 90%, between IHC and qPCR-DMA-Sanger was 83%, and between biochip assay and qPCR-DMA-Sanger was 98%, respectively (Table 6). The frequency of *IDH1* mutations in chondrosarcomas detected by all three methods was 45%, increasing to 53% when a positive result obtained by at least one method was considered. Discrepancies between the methods could be due to the rigorous pretreatment of chondroid tissue before analysis, which may affect the immunogenicity of samples in IHC or degrade DNA in molecular analysis, leading to false negatives in either case. Another reason could be the heterogeneity of the tumor and the use of tissue sections from various areas for different methods. 

In our study, the frequency of *IDH1* mutations differed significantly between tumor types (Table 2 and Table 3). In malignant tumors, *IDH1*-positive cases were predominantly found in 48% of chondrosarcomas, which is consistent with other studies [29,31]. In general, the frequency of *IDH1* mutation in head chondrosarcomas can vary from 0% to 85% depending on the localization, histological type, and stage [8,16,29,30,31]. In our study, two cases of dedifferentiated chondrosarcoma had an *IDH* mutated genotype, defined at least by one method, and no significant difference in *IDH1* mutation frequency between G1 and G2-G3 grades of central chondrosarcoma were found (52% and 51%, respectively). The distribution of *IDH1* mutations by type in our study was close to that described previously: TGT (R132C) accounted for 60% (Table 5) [8].

Clinically, radiologically, and pathomorphologically, chondrosarcoma overlaps with another malignant neoplasm, chordoma, and the differential diagnosis between these tumors presents a major diagnostic challenge [28,35]. Chordomas arise from embryonic remnants of primitive notochord, while chondrosarcomas arise from primitive mesenchymal cells or from the embryonic remnant of the cartilaginous matrix of the skull [36]. Chondrosarcomas treated with similar comprehensive strategies have a significantly better prognosis than chordomas, with overall survival rates of approximately 65% for chordomas and 80% for chondrosarcomas at 5 years, 30% and 50% at 10 years, respectively [37]. 

The main difference between these two chondroid neoplasms is that *IDH1* and *IDH2* mutations, which are frequently found in chondrosarcomas, are not detected in chordomas [6,29,31]. In our study, two *IDH1*-positive cases were found among 15 chordoma specimens, which can be interpreted as misdiagnosed tumors. In addition, one of these cases was negative for brachyury, a transcription factor encoded by the T gene whose expression is highly specific for chordomas (Table 3). On the other hand, tissue decalcification can also lead to a loss of brachyury expression [38]. 

A similar situation was found in osteosarcoma samples, where among seven tumors, two chondroblastic osteosarcomas were also *IDH1* mutation-positive (Table 3). Since *IDH1/2* mutations are uncharacteristic for chordoma and osteosarcoma, these results may request a revision of the diagnosis. In addition, the possibility of false-positive *IDH* mutations should be tested, for example, using different diagnostic approaches.

In the analysis of six benign intracranial chondromas, *IDH1* mutations were detected in 4/6 (66%) (Table 3). However, it should be noted that the sample of chondromas was small, and the results obtained by different methods were inconsistent due to the low quality of tumor material. Chondromas are uncommon intracranial tumors with an estimated incidence rate of 0.2–0.3% of all intracranial tumors [39]. Chondroma malignant degeneration has not been identified, recurrences are rare (5–18%), and so local excision is the treatment of choice. The literature describes that *IDH1/2* mutations are frequently found in central and periosteal chondromas [6], often associated with Ollier’s disease and Maffucci syndrome [7]. These data suggest that chondromas and chondrosarcomas should be considered as being at opposite ends of the pathologic spectrum from benign to malignant tumors, and that somatic *IDH* mutations are suggested to be early events in malignant transformation [40]. Further studies are needed to elucidate this issue. It should be noted that no *IDH1/2* mutations were found in another benign tumor, chondromyxoid fibroma (*n* = 4) (Table 3).

Despite their significant role in the early stages of tumor development, the prognostic value of *IDH* mutations in chondrosarcoma appears to be controversial, with different studies indicating a better [41] or worse [24] prognosis, or no association between *IDH* mutation and outcome [42]. These contradictions may be explained by additional molecular events, which occur during tumor development [13]. It was shown that besides *IDH* mutations; chondrosarcomas often carried mutations in *TP53, CDKN2A/B, COL2A1, YEATS2, NRAS*, and *TERT* genes [13,43,44]. In addition, the overall phenotype of DNA hypermethylation that is characteristic for *IDH*-mutant tumors may change during tumor progression [45]. It is postulated that these genetic and epigenetic events lead to the formation of different molecular subtypes in groups of patients with *IDH*-mutant or *IDH* wild-type chondrosarcomas and affect prognosis and response to treatment. Thus, the complexity of the mutational and epigenetic landscapes must be taken into account when developing new therapeutic strategies for chondroid tumors. 

The limitations associated with this study should be mentioned. The first limitation is related to the peculiarities of FFPE block preparation from chondroid tissues. Aggressive decalcification could lead to deterioration in the quality of tissue samples for IHC staining and degradation of DNA for genetic testing which, in both cases, could affect the results of *IDH1* and *IDH2* mutation analysis. In a number of cases, the small amount of material did not allow for a proper examination by all three methods.

Second, the heterogeneity across tumor samples and the inability to use the same blocks of tumor tissue in different techniques due to the limited amount of biological material could lead to discrepancies in results between methods.

Third, the number of patients in the groups with different neoplasms varied significantly. The most representative group consisted of patients with chondrosarcoma, but the small number of chondroma cases did not allow for a definite conclusion about the *IDH1/2* mutational landscape in this benign tumor with a rare intracranial localization.

Fourth, our study did not investigate the impact of other potential molecular biomarkers that may be associated with *IDH1/2* mutations and influence their diagnostic and prognostic significance. 

## 5. Conclusions

This paper presents a biochip assay for the diagnosis of *IDH1/2* somatic mutations in tumor samples. The main advantages of the method are high sensitivity due to the blocking of wild-type PCR, the possibility of mutation type detection, and simultaneous analysis of *IDH1* and *IDH2* mutations. The method was validated on 96 chondroid tumor samples and showed its efficiency in *IDH1/2* mutation analysis when compared with IHC and real-time PCR with DNA melting analysis using TaqMan probes followed by Sanger sequencing. At the same time, the use of several independent techniques can significantly increase the detection rate of *IDH*-positive samples.

The *IDH1* mutations have been found predominantly in intracranial chondrosarcomas, as well as in chondromas affecting mainly the base of the skull. A further study in a larger sample may be required to determine the frequency of *IDH1/2* mutations in chondromas of the head.

## Figures and Tables

**Table 1:** Clinicopathological characteristics of patients with intracranial chondroid tumors.

Tumor Type	Characteristics	*n* (%)
Chondrosarcoma (*n* = 64)	Mean age, years (range)	41.2 (17–74)
	Sex, female	43 (67.2%)
	Histology (available)	64 (100%)
	Central chondrosarcoma, grade1	19 (29.7%)
	Central chondrosarcoma, grade 2	42 (65.6%)
	Central chondrosarcoma, grade 3	1 (1.6%)
	Dedifferentiated chondrosarcoma	2 (3.1%)
	Tumor location (available)	64
	Cavernous sinus	28 (43%)
	Sphenoid bone	26 (40%)
	Clivus, temporal bone	22 (34%)
	Ethmoid bone	16 (25%)
	Nasal cavity, orbit	14 (21%)
	Chiasmal-sellar region (CSR)	11 (17%)
	Maxillary bone, maxillary sinus	10 (15%)
	Dura mater, occipital bone	6 (9%)
	Falx cerebri	3 (4%)
Chordoma	Mean age, years (range)	37.7 (18–65)
(*n* = 15)	Sex, female	13 (87%)
	Histology (available)	15 (100%)
	Chordoma	8 (53%)
	Chondroid chordoma	5 (33%)
	Dedifferentiated, chordoma + ABC	1 (7%), 1 (7%)
	Tumor location (available)	13
	Clivus	10 (77%)
	Sphenoid bone, cavernous sinus	7 (54%)
	Brachyury (antibodies) (available)	13 (100%)
	Positive	11 (85%)
Osteosarcoma (*n* = 7)	Mean age, years (range)	31.8 (8–72)
	Sex, female	5 (71%)
	Histology (available)	7
	Osteosarcoma, NOS	4 (57%)
	Chondroblastic osteosarcoma	3 (43%)
	Tumor location (available)	7
	Scull base, NOS	7 (100%)
Chondroma (*n* = 6)	Mean age, years (range)	38.6 (31–55)
	Sex, female	5 (83%)
	Tumor location (available)	6
	Superior sagittal sinus	4 (66%)
	Falx cerebri	3 (50%)
	Clivus, sphenoid bone, cavernous sinus	2 (33%)
	Mean age (years, range)	37.5 (27–52)
	Sex, female	4 (100%)
Chondromyxoid	Tumor location (available)	4
	Scull base, NOS	4 (100%)

**Table 2:** Chondrosarcoma cases positive for *IDH1* mutations and determined by any of three methods (NOS—not otherwise specified, ND—not determined, GSR—chiasmal-sellar region; discrepancies in results between methods marked in bold).

ID	Stage	Biochip	qPCR- DMA-Sanger	AA Change	IHC	Tumor Location
1	G1	CTT	CTT	R132L	positive	Cavernous sinus
2	DD	GGT	GGT	R132G	**negative**	Cavernous sinus, clivus
3	G2	TGT	TGT	R132C	positive	Occipital bone
4	G2	CAT	**WT**	R132H	positive	Skull base, NOS
5	G3	TGT	TGT	R132C	positive	Temporal bone
6	G2	GGT	GGT	R132G	positive	Cavernous sinus, clivus
7	G1	**ND**	**WT**	**R132**	positive	Skull base, NOS
8	G2	TGT	TGT	R132C	positive	Skull base, NOS
9	G2	**ND**	**WT**	**R132**	positive	Skull base, NOS
10	G2	TGT	TGT	R132C	positive	Skull base, NOS
11	G2	**ND**	**WT**	**R132**	positive	Clivus, temporal bone
12	G2	TGT	TGT	R132C	positive	Skull base, NOS
13	G2	TGT	TGT	R132C	positive	Dura mater
14	G1	TGT	TGT	R132C	positive	Skull base, NOS
15	G1	AGT	AGT	R132S	**negative**	Cavernous sinu
16	G1	TGT	TGT	R132C	positive	Cavernous sinus
17	G1	TGT	**ND**	R132C	positive	GSR
18	G2	TGT	TGT	R132C	positive	Skull base, NOS
19	G1	TGT	TGT	R132C	positive	Skull base, NOS
20	G1	CTT	CTT	R132L	**ND**	Cavernous sinus
21	G2	CTT	CTT	R132L	positive	Skull base, NOS
22	G2	CTT	CTT	R132L	positive	Craniofacial location
23	G2	**WT**	**WT**	**R132**	positive	Craniofacial location
24	DD	**ND**	TGT	R132C	**negative**	Craniofacial location
25	G2	CTT	CTT	R132L	positive	Skull base, NOS
26	G1	TGT	TGT	R132C	positive	Scull base, NOS
27	G2	TGT	**ND**	R132C	positive	Craniofacial location
28	G2	TGT	TGT	R132C	**negative**	Skull base, NOS
29	G2	TGT	TGT	R132C	**ND**	Cavernous sinus, GSR
30	G2	TGT	TGT	R132C	**negative**	Cavernous sinus, GSR
31	G2	GGT	GGT	R132G	positive	Temporal bone
32	G1	GGT	GGT	R132G	positive	Falx cerebri, dura
33	G2	GGT	GGT	R132G	positive	Cavernous sinus, GSR
34	G2	TGT	TGT	R132C	positive	Ethmoid bone, orbit

**Table 3:** The *IDH1* mutations found in other tumor types (NOS—not otherwise specified, ND—not determined, GSR—chiasmal-sellar region; discrepancies in results between methods marked in bold, CHOS—chondroblastic osteosarcoma, CRD—chordoma, CHND—chondroma).

ID	Tumor Type	Biochip	qPCR- DMA-Sanger	AA Change	IHC	Tumor Location
35	CHOS	**WT**	TGT	R132C	**negative**	Nasopharynx, orbit
36	CHOS	**WT**	**WT**	**R132**	positive	Nasal cavity, orbit, GSR
37	CRD	**WT**	CAT	R132H	positive	Skull base, NOS
38	CRD	AGT	IDH-mut	R132S	**ND**	Cerebellopontine angle
39	CHND	CAT	**ND**	R132H	positive	Superior sagittal sinus
40	CHND	**ND**	TGT	R132C	positive	Skull base, NOS
41	CHND	TGT	**ND**	R132C	**negative**	Falx, superior sagittal sinus
42	CHND	**ND**	TGT	R132C	**negative**	Intracranial space

**Table 4:** Summary of *IDH1* mutation discovery using different approaches.

Genotype	Biochip Assay	qPCR-DMA-Sanger	IHC
All tumors (*n* = 96)			
Determined	(*n* = 89)	(*n* = 88)	(*n* = 84)
Wild-type *IDH1* genotype	57/89 (64%)	56/88 (64%)	53/84 (63%)
Mutated *IDH1* genotype	32/89 (36%)	32/88 (36%)	31/84 (37%)
Chondrosarcomas (*n* = 64)			
Determined	(*n* = 60)	(*n* = 59)	(*n* = 56)
Wild-type *IDH1* genotype	31/60 (51.7%)	32/59 (54%)	29/56 (51.8%)
Mutated *IDH1* genotype	29/60 (48.3%)	27/59 (46%)	27/56 (48.2%)

**Table 5:** Frequency of different nucleotide changes in codon 132 of the *IDH1* gene revealed by molecular methods (nucleotide change marked in bold).

*IDH1* gene (Nucleotide Substitution in Codon 132)	Aminoacid Change	Number of Positive Cases, n (%) (All Tumors, *n* = 37)	Number of Cases, n (%) (Chondrosarcomas, *n* = 30)
CGT > **T**GT	Cysteine (Cys, C)	22 (60%)	18 (60%)
CGT > C**T**T	Leucine (Leu, L)	5 (13.5%)	5 (17%)
CGT > **G**GT	Glycine (Gly, G)	5 (13.5%)	5 (17%)
CGT > C**A**T	Histidine (His, H)	3 (8%)	1 (3%)
CGT > **A**GT	Serine (Ser, S)	2 (5%)	1 (3%)

**Table 6:** Comparison of different approaches to diagnostics of the *IDH1* mutations in chondrosarcomas: biochip assay, qPCR- DMA-Sanger, and IHC.

Results	Biochip vs. IHC (*n* = 51)	Biochip vs. qPCR-DMA- Sanger (*n* = 55)	qPCR- DMA-Sanger vs. IHC (*n* = 50)
Coincidental cases	46/51 (90%)	54/55 (98%)	42/50 (83%)
Non-coincidental cases	5/51 (10%)	1/55 (2%)	8/50 (17%)

## Data Availability

The original contributions presented in the study are included in the article and Supplementary Materials. Further inquiries can be directed to the corresponding author.

## Supplementary Materials

The following supporting information can be downloaded at: https://www.mdpi.com/article/10.3390/diagnostics14020200/s1, Table S1: Primers and TaqMan probes used in real-time PCR with DNA melting analysis title; Table S2: Primers and LNA probes used in biochip assay.
