# Supplementary material for: Diagnostics of IDH1/2 Mutations in Intracranial Chondroid Tumors: Comparison of Molecular Genetic Methods and Immunohistochemistry
Source: Diagnostics (Basel). 2024 Jan 16;14(2):200. doi: 10.3390/diagnostics14020200 (PMC10814347; doi:10.3390/diagnostics14020200)
Supplement: Supplementary file 1 [file diagnostics-14-00200-s001.zip › Supplementary/Table S1.pdf]

Table S1. Primers and TaqMan-probes used in real-time PCR with DNA melting analysis.

**Gene**	**Type**	**Sequence**	**PCR-product**
IDH1	Forward	5'- ATATCCCCCGGCTTGTGAGT-3'	
IDH1	Reverse	5'- ACATACAAGTTGGAAATTTCTGGGC-3'	
IDH1	TaqMan-probe	5'-ROX-CCCATAAGCATGACGACCTATGATGATAG-BHQ2-3'	143 bp
IDH2	Forward	5'-AAACATCCCACGCCTAGTCC-3'	
IDH2	Reverse	5'- AGACAAGAGGATGGCTAGG-3'	
IDH2	TaqMan-probe	5'-ROX-TGGGCGTGCCTGCCAATG-BHQ2-3	148 bp
