# Supplementary material for: Diagnostics of IDH1/2 Mutations in Intracranial Chondroid Tumors: Comparison of Molecular Genetic Methods and Immunohistochemistry
Source: Diagnostics (Basel). 2024 Jan 16;14(2):200. doi: 10.3390/diagnostics14020200 (PMC10814347; doi:10.3390/diagnostics14020200)
Supplement: Supplementary file 1 [file diagnostics-14-00200-s001.zip › Supplementary/Table S2.pdf]

Table S2. Primers and LNA-probes used in biochip assay (LNA-nucleotides are in small letters, P – phosphate group).

**Gene**	**Type**	**Sequence**	**PCR-product length**
IDH1	Forward 1	5’-GCCATTATCTGCAAAAATATCC-3’	
IDH1	Reverse 1	5’-CACATACAAGTTGGAAATTTCTG-3’	160 bp
IDH1	LNA-probe	5’-gCAtgacgaccta-P-3’	
IDH1	Forward 2	5’-AAAATATCCCCCGGCTTGTGA-3’	
IDH1	Reverse 2	5’-TGCAAAATCACATTATTGCCAACAT-3’	105 bp
IDH2	Forward 1	5’- AAACATCCCACGCCTAGTCC-3’	
IDH2	Reverse 1	5’ –TGTGGCCTTGTACTGCAGA-3’	167 bp
IDH2	LNA-probe	5’-gcGTgcctgcCAa-P-3’	
IDH2	Forward 2	5’ –TAGTCCCTGGCTGGACCA-3’	
IDH2	Reverse 2	5’ –GACAAGAGGATGGCTAGG-3’	133 bp
